# Pediatric urinary tract infection: antimicrobial susceptibility profile and resistance-associated factors in a pediatric hospital in São Paulo, Brazil

**DOI:** 10.1590/1984-0462/2026/44/2025173

**Published:** 2026-04-03

**Authors:** Paola Romani Ferreira Suhet, Leticia Correa Marins, Edina Mariko Koga da Silva, Maria Cristina de Andrade

**Affiliations:** a Hospital Infantil Sabará, São Paulo, SP, Brazil.

**Keywords:** Urinary tract infection, Antibiotic resistance, Pediatrics, Infecção urinária, Resistência a antibióticos, Pediatria

## Abstract

**Objective:**

Urinary tract infections (UTIs) are among the most common bacterial infections in childhood and a frequent cause of morbidity and antibiotic use. The aim of this study was to identify urinary pathogens in children and adolescents, assess antimicrobial resistance patterns, and analyze their correlation with clinical factors.

**Methods:**

This observational and retrospective study was conducted in a pediatric hospital from January 1 to December 31, 2022, including patients with positive urine cultures.

**Results:**

A total of 894 cases were analyzed, mostly children aged 1 to 5 years (58.6%) and female (67.9%). *Escherichia coli* was the main pathogen (63.1%), followed by *Proteus mirabilis* (11.1%) and *Pseudomonas aeruginosa* (6.4%). *E. coli* showed high resistance to ampicillin (38.9%) and trimethoprim/sulfamethoxazole (14.5%), maintaining high susceptibility to second- and third-generation cephalosporins and aminoglycosides. Extended-spectrum beta-lactamase was detected in 2.8% of samples. Previous history of UTI was the only factor significantly associated with antimicrobial resistance.

**Conclusions:**

The findings underscore the importance of continuous epidemiological surveillance to guide empirical treatment and rational antibiotic use in children.

## RESUMO

**Objetivo:**

As infecções do trato urinário se caracterizam como as infecções bacterianas mais comuns na infância e representam uma causa frequente de morbidade e uso de antibióticos. Este estudo teve como objetivo identificar os uropatógenos responsáveis por infecções do trato urinário em crianças e adolescentes, avaliar os padrões de resistência antimicrobiana e analisar sua correlação com fatores clínicos.

**Métodos:**

Estudo observacional e retrospectivo conduzido em um hospital pediátrico entre 1º de janeiro e 31 de dezembro de 2022, incluindo pacientes com uroculturas positivas.

**Resultados:**

Foram analisados 894 casos, a maioria entre 1 e 5 anos (58,6%) e do sexo feminino (67,9%). A *Escherichia coli* foi o principal patógeno (63,1%), seguida por *Proteus mirabilis* (11,1%) e *Pseudomonas aeruginosa* (6,4%). Observou-se alta resistência da *Escherichia coli* à ampicilina (38,9%) e ao trimetoprima/sulfametoxazol (14,5%), com boa suscetibilidade às cefalosporinas de segunda e terceira geração e aos aminoglicosídeos. Um total de 2,8% das amostras foi positivo para beta-lactamase de espectro estendido. A infecção do trato urinário prévia foi o único fator significativamente associado à resistência antimicrobiana.

**Conclusões:**

Os achados reforçam a importância da vigilância epidemiológica para orientar o tratamento empírico e o uso racional de antibióticos na infância.

## INTRODUCTION

 Urinary tract infections (UTIs) are among the most prevalent bacterial infections in childhood, representing a leading cause of pediatric medical consultations, hospitalizations, and antibiotic prescriptions.^1^ According to the literature, approximately 8% of girls and 2% of boys will experience at least one episode of UTI by the age of 7, and nearly 30% of children will have a recurrence within 1 year of the initial episode.^2^ Although most UTIs are treatable, they remain a significant public health concern due to their potential to cause long-term complications such as renal scarring, hypertension, and advanced stages of chronic kidney disease, especially in cases of recurrent infections or those associated with structural abnormalities of the urinary tract.^1^

 Diagnosing UTIs in pediatrics poses a clinical challenge that requires integrating clinical, laboratory, and epidemiological criteria. According to international guidelines such as those from the National Institute for Health and Clinical Excellence (NICE, 2007) and the American Academy of Pediatrics (AAP, 2011), the diagnosis is confirmed by the presence of pyuria along with a single pathogen growing at a concentration of at least 50,000 colony-forming units (CFUs)/mL in a urine sample collected via bladder catheterization. For toilet-trained children, the clean-catch midstream method is standard, while catheterization is preferred in younger children. Although widely used, urine collection via adhesive perineal bags is associated with a high false-positive rate and is therefore more appropriate for initial screening.^3,4^

 The etiology of pediatric UTIs is predominantly bacterial, with *Escherichia coli* (*E. coli*) accounting for approximately 80–90% of community-acquired cases.^1,5^ Other frequently isolated pathogens include *Proteus mirabilis*, *Klebsiella pneumoniae*, and *Pseudomonas aeruginosa*. The accurate identification of the etiologic agent is essential for selecting the most appropriate antibiotic therapy, particularly in recurrent or complicated infections. However, the growing antimicrobial resistance, especially among *E. coli* and other Enterobacteria, has compromised the effectiveness of traditional empiric treatments such as ampicillin and trimethoprim/sulfamethoxazole.^6^

 This scenario is further complicated by the emergence of extended-spectrum beta-lactamase (ESBL)-producing strains, which confer resistance to third-generation cephalosporins and monobactams. These strains are associated with increased morbidity, higher hospitalization rates, and the need for carbapenems as last-resort therapy.^7^ Recent studies have reported a rising prevalence of ESBL-producing pathogens even in community-acquired infections, highlighting the need for continuous surveillance and therapeutic strategies informed by local resistance patterns.^8^

 Given this context, the present study aims to contribute to understanding antimicrobial resistance patterns and associated factors in pediatric UTIs among patients treated at a tertiary pediatric hospital in São Paulo, Brazil. Analysis of isolated pathogens, antimicrobial susceptibility profiles, and the prevalence of ESBL-producing strains will help identify local resistance trends, support the adaptation of treatment guidelines, and promote more effective, individualized infection management. 

## METHOD

 This retrospective observational study analyzed electronic medical records (EMRs) of pediatric patients who presented with positive urine cultures between January 1 and December 31, 2022. Data were collected from the EMR system of a private quaternary pediatric hospital located in São Paulo, Brazil, which exclusively provides healthcare services to children. 

 Initially, all EMRs from 2022 reporting a positive urine culture were identified. Each record was then manually reviewed, and relevant demographic, clinical, and microbiological data were extracted and organized into a standardized electronic database. The inclusion criteria encompassed patients from birth to under 18 years of age, who were seen in the emergency department, inpatient wards, or intensive care unit, and who had a positive urine culture defined as ≥50,000 CFUs of a single uropathogen. For cases where urine was collected by midstream cleancatch, inclusion required documentation of urinary symptoms and pyuria. Patients diagnosed with asymptomatic bacteriuria, those with urine samples collected externally or not evaluated by the hospital’s medical team, and patients already hospitalized for other reasons were excluded. To avoid duplicate entries, only one infection episode per patient was included within any 30-day period. 

 For the interpretation of antimicrobial susceptibility results, both "susceptible" (S) and "susceptible with increased exposure" (I) categories were considered susceptible, while the "resistant" (R) category was maintained as resistant, following EUCAST/BrCAST guidelines. 

 Statistical analysis was performed using standard statistical tests. In all analyses, the main outcome was antimicrobial resistance of the uropathogen, categorized as resistant ("yes") or non-resistant ("no"). Categorical variables were compared between patients with and without antimicrobial resistance using the ꭓ^2^ test or Fisher’s exact test, as appropriate. Continuous variables were compared between these two groups using the Student’s t-test for independent samples. A p<0.05 was considered statistically significant. 

 The Institutional Research Ethics Committee approved this study under protocol number 6.450.026 and CAAE 74674023.0.0000.5567. Given the study’s retrospective nature and the use of anonymized data, informed consent was waived. 

## RESULTS

**Figure 1:**
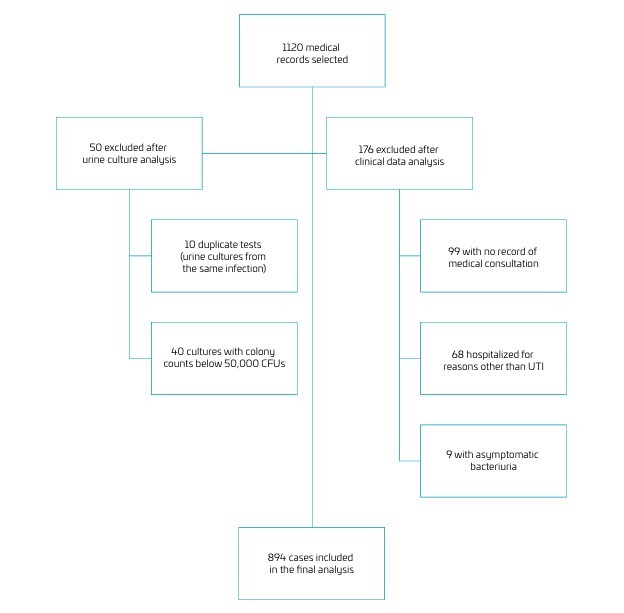
Schematic representation of electronic EMR selection. Illustrates the selection process of EMRs. Of the 1120 records initially screened, 50 were excluded based on urine culture criteria, and 176 were excluded after clinical data analysis. A total of 894 cases were included in the final analysis. Abbreviations: EMR: electronic medical record; CFU: colony-forming unit; UTI: urinary tract infection.

 The final sample, after applying the inclusion and exclusion criteria, comprised 894 individual infection episodes (Figure 1). Most patients were between 1 and 5 years of age (58.6%) and female (67.9%). *E. coli* was the most frequently isolated pathogen (63.1%), followed by *P. mirabilis* (11.1%), *P. aeruginosa* (6.4%), *K. pneumoniae* (4.5%), *Enterococcus faecalis* (4.4%), and other agents (10.3%) (Table 1). Only 4.4% of patients were on antibiotic prophylaxis, most commonly with cephalexin (36.8%) or trimethoprim/sulfamethoxazole (34.2%). 

**Table 1:** Baseline demographic and clinical characteristics of the study population. Describes patient age, sex, antibiotic prophylaxis use, type of prophylactic agent, and distribution of uropathogens identified.

		n	%
Age			
	0 to 12 months	149	16.7
	1 to 5 years	524	58.6
	Over 5 years	221	24.7
Sex			
	Female	607	67.9
	Male	287	32.1
Antibiotic prophylaxis			
	No	848	95.6
	Yes	39	4.4
Type of antibiotic prophylaxis			
	Cephalexin	14	36.8
	Trimethoprim/sulfamethoxazole	13	34.2
	Nitrofurantoin	6	15.8
	Macrodantine	5	13.2
Pathogens			
	Escherichia coli	564	63.1
	Proteus mirabilis	99	11.1
	Pseudomonas aeruginosa	57	6.4
	Klebsiella pneumoniae	40	4.5
	Enterococcus faecalis	39	4.4
	Others	95	10.3

 When factors associated with antimicrobial resistance were analyzed, a previous history of UTI was the only variable significantly associated with resistance. A prior UTI was more frequent among children with resistant isolates compared with those with non-resistant isolates (31.0% vs 23.7%; p = 0.022) (Table 2). No significant associations were observed between resistance and urological abnormalities (such as neurogenic bladder, posterior urethral valve, or other urinary tract malformations), constipation, or antibiotic prophylaxis. Among patients who had received antibiotics in the preceding 12 months, there was no significant difference in the mean number of antibiotic courses between the non-resistant group (mean 1.24 courses, standard deviation 0.34) and the resistant group (mean 1.34 courses, standard deviation 0.69; p=0.121). 

**Table 2:** Relationship between antimicrobial resistance and the distribution of baseline variables. ꭓ^2^ test was used to analyze associations between antimicrobial resistance and underlying clinical conditions, history of previous urinary tract infection, and use of antibiotic prophylaxis.

Resistance		Yes		No		p-value
		n	%	n	%	
Neurogenic bladder						
	No	350	98.3%	512	99.4%	0.214
	Yes	6	1.7%	3	0.6%	
Ureteropelvic junction obstruction						
	No	356	100%	513	99.6%	0.648
	Yes	0	0.0%	2	0.4%	
Posterior urethral valves						
	No	353	99.1%	514	99.8%	0.379
	Yes	3	0.9%	1	0.2%	
Other urinary tract malformations						
	No	324	99%	482	98.3%	0.196
	Yes	32	1%	33	0.7%	
Constipation						
	No	337	94.3%	484	93.6%	0.781
	Yes	19	5.6%	31	6.4%	
Previous history of UTI						
	No	243	55.1%	383	69%	0.022
	Yes	109	44.9%	119	31%	
Antibiotic prophylaxis						
	No	334	94.3%	493	96.1%	0.312
	Yes	19	5.7%	19	3.9%	

UTI: urinary tract infection.

 Considering all urinary pathogens together, the highest resistance rates were observed for ampicillin (34.2%), ceftazidime/avibactam (17.7%), ceftazidime (13.3%), and trimethoprim/sulfamethoxazole (11.2%). Resistance to other antibiotics was generally below 10%, including ceftriaxone (8.0%), amoxicillin/clavulanate (6.0%), gentamicin (8.3%), and nitrofurantoin (4.0%). Low resistance rates were seen in vancomycin (0.5%) and meropenem (3.6%). 

 Among the 894 positive urine cultures, *E. coli* was isolated in 564 samples. In this subgroup, resistance was 38.9% to ampicillin and 14.5% to trimethoprim/sulfamethoxazole, while resistance to cefuroxime, ciprofloxacin, and amoxicillin/clavulanate remained below 7% (Table 3). *E. coli* showed high susceptibility to second- and third-generation cephalosporins (cefuroxime 94.6% and ceftriaxone 93.9%), aminoglycosides (amikacin 100%), nitrofurantoin (94.7%), and quinolones (ciprofloxacin 93.8% and norfloxacin 97.6%). 

**Table 3:** Resistance profile of *Escherichia coli*. Shows the resistance rates of *E. coli* isolates to commonly tested antibiotics.

	Resistance	
	n	%
Amikacin	0	0.0
Amoxicillin/clavulanate	24	4.5
Ampicillin	177	38.9
Cefepime	5	4.8
Ceftazidime/avibactam	6	14.0
Ceftriaxone	25	6.1
Cefuroxime	15	5.4
Ciprofloxacin	9	6.3
Clindamycin	3	7.0
Ertapenem	13	5.6
Gentamicin	23	7.4
Levofloxacin	14	5.6
Meropenem	0	0.0
Nitrofurantoin	7	5.3
Norfloxacin	5	2.4
Oxacillin	3	1 .9
Piperacillin/tazobactam	4	1.3
Polymyxin B	12	5.5
Tobramycin	15	10.4
Trimethoprim/sulfamethoxazole	19	14.5
Vancomycin	1	0.7

 ESBL production was detected in 25 urine samples (2.8%). *E. coli* accounted for 16 of these isolates (64%), followed by *K. pneumoniae* in 7 (28%), *Enterobacter cloacae* in 1 (4%), and *Proteus penneri* in 1 (4%). Most patients with ESBL-producing organisms were between 2 and 10 years of age (52%), while 32% were younger than 2 years and 16% were older than 10 years. Approximately half of these children (52%) had no documented comorbidities. Among those with associated conditions, we observed chronic non-progressive encephalopathy and other genetic syndromes in 12% each, neurogenic bladder and functional constipation in 8% each, and other urinary tract malformations and posterior urethral valve in 4% each. 

 Regarding prior infectious history, 20% of patients with ESBL-positive samples reported at least one prior UTI, and 60% had already been hospitalized for treatment. In the 12 months preceding the ESBL episode, 58% had received antibiotics such as amoxicillin/clavulanate, ceftriaxone, ampicillin, or gentamicin. During the current episode, 36% of ESBL cases required hospitalization. The resistance profile of ESBL-producing strains is detailed in Table 4. 

**Table 4:** Resistance profile of extended-spectrum beta-lactamase -producing bacteria. Displays the distribution of extended-spectrum beta-lactamase -positive organisms by species, based on 25 isolates.

	Resistance	
	n	%
Amoxicillin/clavulanate	23/25	92
Cefepime	24/25	96
Ceftriaxone	25/25	100
Cefuroxime	25/25	100
Gentamicin	14/25	56
Ciprofloxacin	14/25	56
Trimethoprim/sulfamethoxazole	21/25	84
Meropenem	2/25	8
Ertapenem	2/25	8

## DISCUSSION

 In this retrospective cohort of 894 pediatric UTIs seen at a tertiary hospital in São Paulo, most cases occurred in girls aged 1–5 years, and *E. coli* was the predominant uropathogen, followed by *P. mirabilis* and *P. aeruginosa*. *E. coli* showed high resistance to ampicillin and lower, but still relevant, resistance to trimethoprim/sulfamethoxazole, whereas susceptibility to second- and third-generation cephalosporins, aminoglycosides, and nitrofurantoin remained high. ESBL-producing strains were identified in 2.8% of samples, mostly *E. coli* and *K. pneumoniae*. A previous history of UTI was the only clinical factor significantly associated with antimicrobial resistance. 

 The distribution of etiologic agents in our cohort is consistent with the classical epidemiology of pediatric UTIs, in which *E. coli* accounts for most community-acquired infections, while other Enterobacterales and *P. aeruginosa* are less common, often in more complex or healthcare-associated settings.^1,5,9,10^ The predominance of *E. coli* above 60% and the lower frequencies of *P. mirabilis*, *K. pneumoniae*, and *P. aeruginosa* in our study mirror findings from Brazilian and international series, reinforcing the external validity of our data for similar pediatric settings.^1,5,10^

 Regarding antimicrobial susceptibility, our results confirm the progressive loss of efficacy of ampicillin and, to a lesser extent, trimethoprim/sulfamethoxazole for empirical treatment of pediatric UTIs. Previous studies have reported *E. coli* resistance rates to ampicillin ranging from 50% to 70% and to trimethoprim/sulfamethoxazole above 40%, while maintaining high susceptibility to cephalosporins, aminoglycosides, and nitrofurantoin.^2,5,10-13^ In our cohort, *E. coli* resistance to ampicillin (38.9%) and trimethoprim/sulfamethoxazole (14.5%) was comparatively lower than in many of these reports, but still sufficiently high to discourage their empirical use as first-line monotherapy. Conversely, their excellent susceptibility to second- and third-generation cephalosporins, aminoglycosides, and nitrofurantoin supports their role as preferred options, particularly given patient age, clinical severity, and local stewardship policies. 

 An important finding of this study was the association between previous UTI and antimicrobial resistance. Children with recurrent infections were more likely to harbor resistant organisms, including ESBL-producing strains, than those with a first episode. This observation is in line with evidence that repeated antibiotic exposure and persistent urinary tract colonization select for resistant organisms and foster bacterial persistence in the urinary tract.^11,13^ These results underscore the need for appropriate early treatment and judicious antibiotic use to curb resistance, reduce morbidity, and preserve therapeutic options. Preventive strategies targeting recurrent UTIs may play a central role in mitigating resistance in pediatric populations. 

 In our cohort, only a small proportion of patients were under antibiotic prophylaxis, and we did not observe a statistically significant association between prophylaxis and overall resistance (p=0.312). However, nearly half of the children on trimethoprim/sulfamethoxazole prophylaxis (6/13) developed resistance to this drug during the UTI episode, whereas no nitrofurantoin-resistant isolates were documented among those using nitrofurantoin prophylaxis. These findings are consistent with meta-analyses suggesting that long-term prophylaxis, particularly with trimethoprim/sulfamethoxazole, may increase resistance. At the same time, nitrofurantoin seems to exert a lower selective pressure. However, adherence and tolerability issues must also be considered, as the apparent benefit may reflect poor adherence due to side effects or palatability issues, thereby reducing selective pressure.^14,15^ Future studies evaluating adherence and behavioral factors are warranted to better understand the realworld impact of prophylactic strategies. The optimal agent for prophylaxis remains undefined, and individualized approaches are recommended, prioritizing low-resistance potential and aligning with local epidemiology. Additionally, decisions should consider underlying urological anomalies and prior UTI history. 

 ESBL-producing Enterobacteriaceae accounted for a minority of isolates in this series, yet their clinical relevance is considerable. The prevalence of 2.8% is comparable to that reported in recent pediatric cohorts. Yet, these strains exhibited very high resistance rates to third- and even fourth-generation cephalosporins, as well as to amoxicillin/clavulanate and trimethoprim/sulfamethoxazole, limiting empirical therapeutic options.^6-8,16,17^ In contrast, susceptibility to carbapenems remained preserved, reinforcing their role as last-resort agents for severe infections. The presence of ESBL-producing organisms in community-acquired infections in children, often in the context of previous antibiotic exposure or urogenital anomalies, highlights the need for continuous local surveillance and for empiric regimens guided by current resistance profiles. 

 This study has limitations inherent to its retrospective design and reliance on data from a single center, which may limit the generalizability of the findings. Clinical outcomes such as recurrence, treatment failure, and long-term renal sequelae were not systematically evaluated and therefore could not be correlated with the microbiological profiles. In addition, some clinical variables may have been underreported or inconsistently documented in the medical records, potentially attenuating associations with resistance. 

 Despite these limitations, our data provide a robust contemporary overview of the etiologic agents and antimicrobial resistance patterns of pediatric UTIs in a tertiary pediatric Brazilian hospital, emphasizing the importance of local epidemiological surveillance to support empirical treatment and antibiotic stewardship strategies. 

 In conclusion, in this cohort of children and adolescents with UTI, *E. coli* was the main uropathogen and showed high resistance to ampicillin and moderate resistance to trimethoprim/sulfamethoxazole, while susceptibility to second- and third-generation cephalosporins, aminoglycosides, and nitrofurantoin remained high. Previous history of UTI was the only clinical factor significantly associated with antimicrobial resistance. These findings reinforce the need for continuous local surveillance and for empirical antibiotic regimens guided by current resistance patterns to promote rational antibiotic use in pediatrics. 

## Data Availability

The database that originated the article is available with the corresponding author.
